# The Role of m6A Epigenetic Modification in the Treatment of Colorectal Cancer Immune Checkpoint Inhibitors

**DOI:** 10.3389/fimmu.2021.802049

**Published:** 2022-01-06

**Authors:** Huan Tong, He Wei, Alhaji Osman Smith, Juan Huang

**Affiliations:** ^1^ Department of Hematology, Sichuan Academy of Medical Sciences & Sichuan Provincial People’s Hospital, University of Electronic Science and Technology of China, Chengdu, China; ^2^ Blood Diseases Institute, Xuzhou Medical University, Xuzhou, China & Department of Hematology, The Affiliated Hospital of Xuzhou Medical University, Xuzhou, China & Key Laboratory of Bone Marrow Stem Cell, Xuzhou, China; ^3^ Department of Gastroenterology, The Second Affiliated Hospital of Chengdu Medical College, Nuclear Industry 416 Hospital, Chengdu, China; ^4^ School of Bioscience and Technology, Chengdu Medical College, Chengdu, China

**Keywords:** colorectal cancer, ICB, resistance, m6A epigenetics, overcome resistance

## Abstract

Tumor immunotherapy, one of the efficient therapies in cancers, has been called to the scientific community’s increasing attention lately. Among them, immune checkpoint inhibitors, providing entirely new modalities to treat cancer by leveraging the patient’s immune system. They are first-line treatments for varieties of advanced malignancy, such as melanoma, gastrointestinal tumor, esophageal cancer. Although immune checkpoint inhibitors (ICIs) treatment has been successful in different cancers, drug resistance and relapses are common, such as in colorectal cancer. Therefore, it is necessary to improve the efficacy of immune checkpoint therapy for cancer patients who do not respond or lowly response to current treatments. N6-methyladenosine (m6A), as a critical regulator of transcript expression, is the most frequently internal modification of mRNA in the human body. Recently, it has been proposed that m6A epigenetic modification is a potential driver of tumor drug resistance. In this report, we will briefly outline the relevant mechanisms, general treatment status of immune checkpoint inhibitors in colorectal cancer, how m6A epigenetic modifications regulate the response of ICIs in CRC and provide new strategies for overcoming the resistance of ICIs in CRC.

## Introduction

The 2018 Global Cancer Statistics estimates the incidence and mortality of 36 cancers in 185 countries around the world. It is reported that the incidence of colorectal cancer is 6.1%, and the mortality rate is 9.2%. The incidence and mortality rate of colorectal cancer is second only to breast cancer in women. In contrast, the incidence and mortality of colorectal cancer in men are second only to lung cancer and prostate cancer (1). In short, the incidence and mortality of colorectal cancer are currently at a high position, which should be paid great attention to and urgently needed effective measures to reduce. In the previous low-incidence areas, the incidence of colorectal cancer showed an upward trend (2). Recently, it has been suggested that the patterns and trends of the incidence and mortality of colorectal cancer are connected to human beings’ current living standards and lifestyles (3). While China’s economic situation is now changing from developing countries to developed countries, Chinese average living standards get remarkably improved. Simultaneously, China is currently also in the stage of cancer transformation, the cancer spectrum is changing toward developed countries, and the cancer burden of colorectal cancer is increasing rapidly (4). Therefore, it is essential to prevent the onset of colorectal cancer and cure colorectal cancer patients, then to improve people’s quality of life.

The current management strategies for colorectal cancer mainly include surgery, chemotherapy, radiotherapy, and immunotherapy (5, 6). Among them, cancer immunotherapy, containing active immunotherapy, passive immunotherapy, and immune checkpoint inhibitors, is gradually becoming the mainstream of modern cancer treatment (7), which has become a new research direction for cancer treatment and has received extensive attention (8, 9).

Recently, tumor immunotherapy, especially in immune checkpoint blockade treatment, has achieved remarkable success in the treatment of colorectal cancer. In particular, immune checkpoint inhibitors have been used in mismatch repair defects and high microsatellite instability (dMMR – MSI-H) metastasis, which have been shown to be effective in patients with colorectal cancer and have been approved by the FDA, such as pembrolizumab, nivolumab. Compared with most other treatments for malignant metastatic cancer, immune checkpoint blockade achieved long-term and durable remission in some patients, highlighting the excellent prospects of immune checkpoint blockade in treating colorectal cancer (10–14).

However, with the significant improvement of people’s living standards, the quantity of patients with colorectal cancer has continued to hoist, increasing treatment difficulties are found nowadays. It is currently believed that only a tiny proportion of patients with colorectal cancer respond to immune checkpoint inhibitors treatment. While most proficient in mismatch repair (pMMR) and microsatellite stabilization (MSS) or low levels tumors with microsatellite instability (MSI-L) (called pMMR-MSI-L tumors) are ineffective. In these tumors, low tumor mutation burden and lack of immune cell infiltration are thought to be mechanisms of immune resistance (15, 16).

Because most pMMR and MSS or MSI-L patients with colorectal cancer is ineffective in ICIs, we wonder that, whether there exist some deeply resistant mechanisms in these patients. It has been proposed that epigenetic modification is a potential driver of tumor drug resistance (17). Since epigenetic inheritance is reversible in nature, the strategy of reversing epigenetic abnormalities is considered to be helpful in treating cancer and reversing drug resistance in cancer therapy (18–20).

In this report, we will briefly outline the relevant mechanisms of tumor immunity, the possible mechanism of tumor ICIs in treating tumors, the current status of colorectal cancer immune checkpoint inhibitors, and a potential method of m6A epigenetic modifications to regulate colorectal cancer ICIs response.

## Immunological Mechanisms for Cancer

In human immune system is divided into three categories: the immune defense, immune surveillance, and immune stability, in which tumorigenesis is closely related to the immune surveillance function in the body. Specifically, when the body discovers some cells become cancerous, the immune system will develop an innate immune response and an adaptive immune response targeting to tumor cells or antigens. These immune effector mechanisms influence and regulate each other to achieve the removal of tumor cells within the body (21–25).

**Figure 1:**
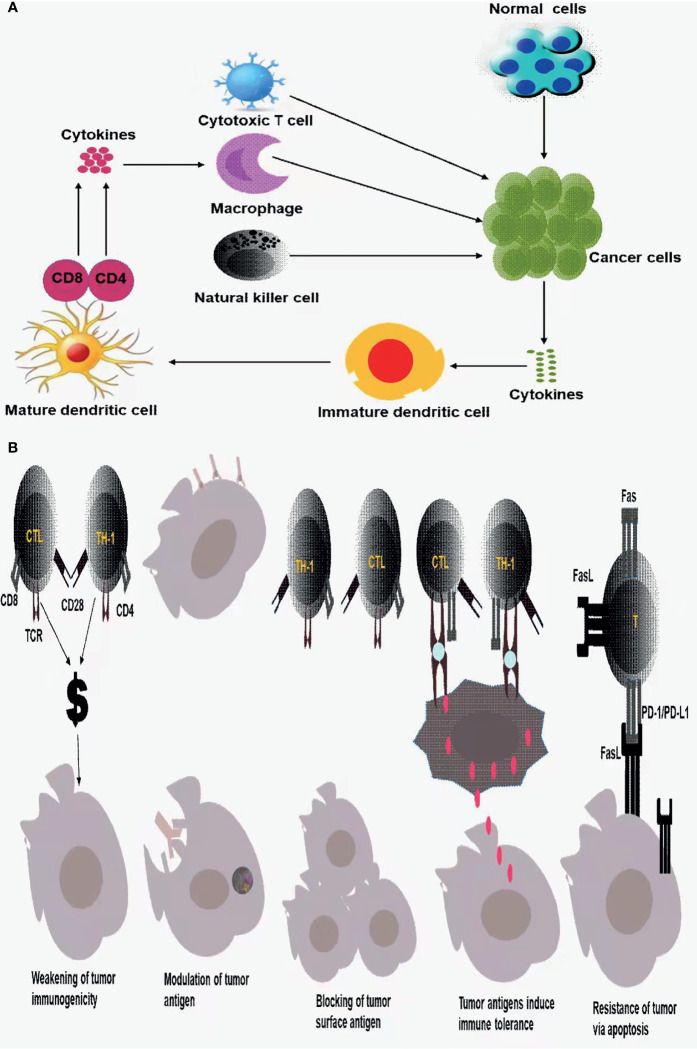
**(A)** Innate immune response and adaptive immune response to tumor cells; **(B)** tumor immune escape mechanism.

In this process, cancer cells secrete some cytokines, which will stimulate the maturation of immature antigen-presenting cells, such as dendritic cells. Mature antigen-presenting cells then present relevant antigens to CD4+ or CD8+ T cells to make them respond, such as secreting some cytokines, which can further act on cytotoxic lymphocytes, Natural killer Cells, macrophages, and enhance the killing of cancer cells, as shown in **Figure 1A**.

However, even due the body has a series of intelligent immune surveillance, immune clearance of tumor cells, tumor cells escape the body’s immune response and weaken the immune system, that result in tumor immune escape mechanism (21, 25).

Regarding the tumor immune escape mechanism, the current standard views are as follows: a. The immunogenicity of tumor cells is weakened or missing, b. Tumor antigen modulation, that is, due to the body’s immune response to tumor antigens, tumor cell surface antigens are reduced, weakened, or disappear, thus leaving the immune system unrecognizable, allowing the tumor cells to escape the host immune attack, c. tumor cell surface antigens are covered or blocked, d. tumor antigens induce immune tolerance, e. tumor cells would resist apoptosis and induce immune cell apoptosis through Fas/FasL, PD-1/PD-L1 pathways, tumor cells induce immune suppression (21, 25, 26) as displayed in **Figure 1B**.

## Tumor Immune Checkpoint Inhibitors

Given that the body’s immune system plays a vital role in the development and progression of tumors, tumor immunotherapy utilizing the body’s own immune system to fight against tumor cells is booming. Immunotherapy is expected to become a new development following surgery, chemotherapy, radiotherapy, and targeted tumor therapy as a generation of tumor treatment methods (5, 6). The goal of tumor immunotherapy is to release the negative regulatory mechanism in the body and then enhance the immune response of cancer immunity (27). A typical example is to block PD-1/PD-L1 or CTLA-4.

**Figure 2:**
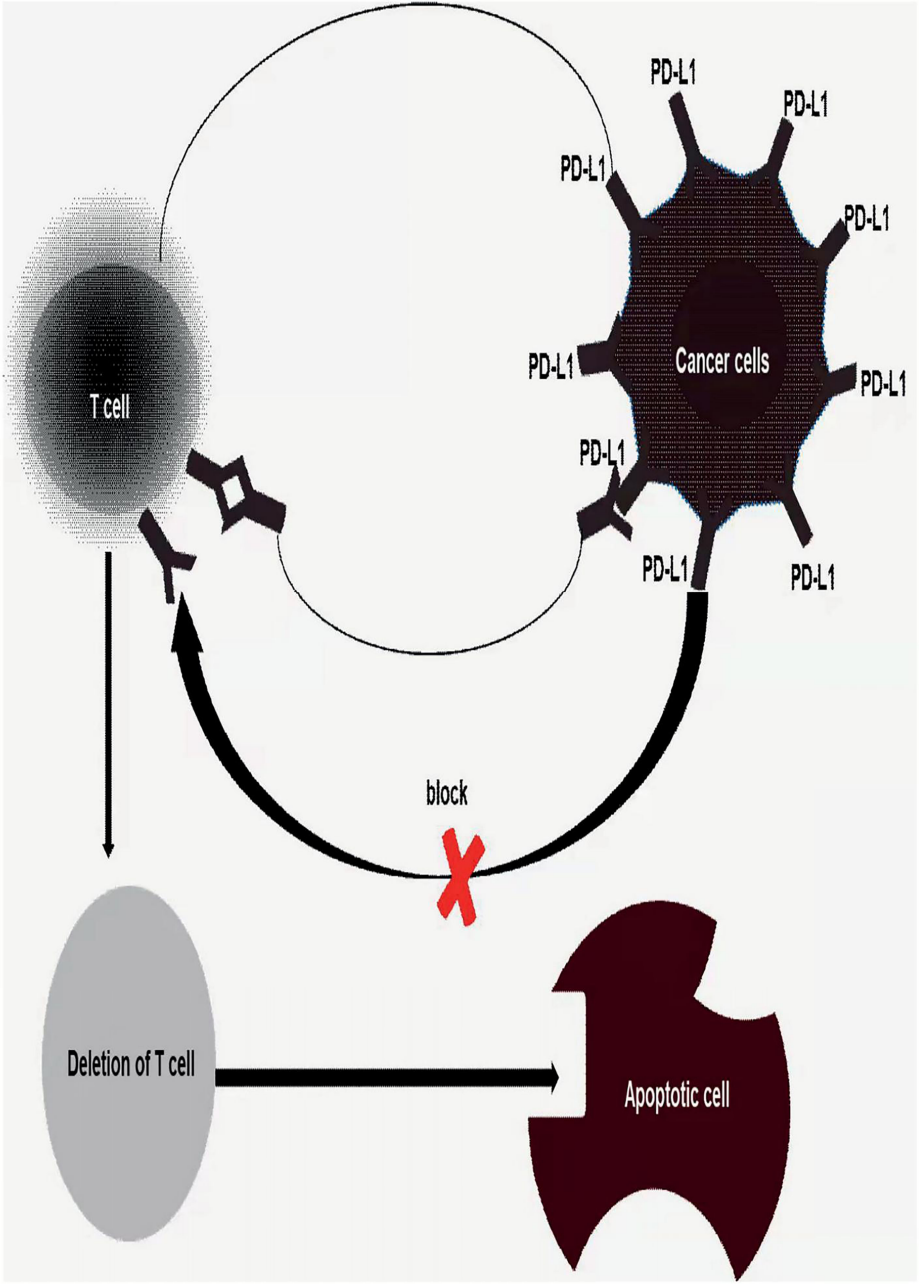
Once tumor’s PD-L1 binds to T cells’s PD-1 induced depletion, disability and apoptosis of T cells, achieves tumor immune escape. Thus, block the binding of PD1/PD-L1 would be an effective treatment for human cancer or inhibiting tumor proliferation and metastasis.

Both PD-1 and CTLA-4 are checkpoints that co-inhibit signaling, which controls T-cell activities, such as activation, proliferation. Immune checkpoint inhibitors, such as PD-1/PD-L1 inhibitors and CTLA-4 inhibitors, is a way to remove tumor cells from the inhibitory effect of tumor cells on the immune system, release immune responses, and eliminate tumor cells, which are the excellent content of immunotherapy for colorectal cancer. On the surface of natural killer cells, dendritic cells, B cells,T cells, macrophages, MDSCs, et al. there exists substantial receptors—PD-1(PDCD1/CD279) (28–30). However, PD-1 is usually not expressed in inactivated T cells. PD-1 are not highly expressed until T cells are activated. The ligand—PD-L1/PD-L2, is a transmembrane protein that can bind to PD-1 and then negatively regulate T cell function, specifically, T cells’ activation, proliferation, and survival (31). In tumor microenvironment, tumor cells and tumor-associated APCs highly express PD-L1, while tumor-infiltrating lymphocytes are gradually rich in PD-1 expression under long-term stimulation of tumor antigens. The combination of PD-L1 and PD-1 can induce T cell apoptosis, disability, and depletion (**Figure 2**), thereby inhibiting the activation, proliferation, and anti-tumor function of tumor antigen-specific CD8+ T cells and achieving tumor immune escape (32–39). Noticeably, the use of PD-1/PD-L1 inhibitors would block the PD-1 signaling pathway, restore the body’s tumor immunity to normal, and eliminate tumor cells in the body.

CTLA-4(CD152), is a transmembrane protein, whose receptors are B7-1(CD80) and B7-2(CD86). CTLA-4 and CD28 share the same receptor, but each of them in the body plays a completely different role. Triggering CD28 will strongly up-regulate T cells function, enhancing T cell activation and cytokine production, while triggering CTLA-4 wouldn’t not only produce the same effect, but may also down-regulate CD28-mediated effects. Generally speaking, CD28 is expressed on resting and activated T cells while CTLA-4 is only expressed on activated T cells. Furthermore, CTLA-4 has a stronger affinity for B7 molecules than CD28 (40). After the TCR-CD3 complex is formed, CTLA-4 will rapidly up-regulate and bind with its high affinity to the co-activated receptors CD80 and CD86 expressed on antigen-presenting cells, thus negatively regulates the activation and function of T cells (41–47). In the tumor microenvironment, the expression of CTLA-4 in tumor infiltrating regulatory T cells (Treg) is increased (38, 48–50), thereby inhibiting the activation of tumor antigen-specific T cells, proliferation and anti-tumor function, realize tumor escape. Unlike PD-1/PD-L1 inhibitors, the using of CTLA-4 inhibitors would like to relieve the inhibitory effect of regulatory T cells (Treg) in the tumor microenvironment, enhancing the body’s tumor immunity to eliminate tumor cells in the body.

Inhibition of PD1/PD-L1 and/or CTLA, beyond all doubt, would become an effective therapy to treat human cancers including colorectal cancer or to inhibit tumor proliferation and metastasis.

## ICIs Treatment for Colorectal Cancer

Starting from this mechanism of tumor immune escape, inhibit the binding of tumor PD-L1 and T cell surface PD-1, block negative regulatory signals, restore the normal immune response of T cells to tumor cells to realize the role of removing tumor cells. Based on this mechanism, a large number of colorectal cancer patients have now been treated with ICIs. Fortunately, in treating colorectal cancer patients with PD-1, PD-L1, and CTLA-4 inhibitors, patients with high microsatellite stability (MSI-H) and mismatch repair defects showed fine efficacy (10–14, 51, 52). Recent researches demonstrated that Cytotoxic T cells, memory T cells, Th1 cells, N.K. Cells, TFH cells, B cells, activated and mature D.C., M1 macrophages, FOXP3^1ow^CD45RA- Treg cells, high PD-L1 expression on the exterior of immune cells were confirmed to be strongly associated with a favorable prognosis of CRC (53–59), while CRC patients with massive infiltration of FOXP3^hi-^ Treg cells, Th17 cells, M2 macrophages, MDSCs, LAMP3 DC and neutrophils have generally a poor prognosis (60–63). Therefore, by raising the level of cells associated with good prognosis of colorectal cancer or lowering the level of cells associated with poor prognosis of colorectal cancer, it may improve the efficacy of colorectal cancer patients. Besides, one of the evaluation indicators over ICIs treatment response is the quantification of circulating tumor DNA (ctDNA). As a practical predictive approach, this indicator can be used in early monitoring to assess the tumor response of patients receiving anti-PD-1 therapy (64). Furthermore, it has shown that MSI, tumor mutation burden(TMB), and MMR played a critical role in the ICB response of colorectal cancer. Generally speaking, CRC patients with MSI-H, high TMB, and dMMR are considered to have a positive reaction to ICIs treatment. Thus CRC patients can get a considerably brilliant prognosis (65–67).

In 2010, Julie R Brahmer, et al. first reported anti-PD-1 in 39 patients with different advanced maglicies, in which one CRC receiving durable complete remission, while other cancers, like melanoma, renal cell cancer did not meet the PR criteria but had significant tumor lesion regression (68). With the in-depth study of anti-immune checkpoint treatment of cancer subsequent studies showed significant activity of PD-1 blockers in multiple cancers, leading to clinical approval for the treatment of multiple tumors, such as gastric cancer (69), colorectal cancer (70), liver cancer (71), and pancreatic cancer (72–74). However, despite of abundant success in treating CRC, there still exists resistance to ICIs in colorectal cancer. The results of current clinical studies are quite explorable, which encourage us to further explore the underlying mechanism of non-response or low-response to ICIs for CRC patients.

Thus, varieties of researches including basic and clinical are done over enhancing CRC efficiency. Researchers made a conclusion that CRC patients with MSI-H, high TMB and dMMR are demonstrated to get a nice response to ICIs treatment and own a considerably brilliant prognosis (65–67, 75–77). Following, FDA approved pebrolizumab used for the treatment of refractory, metastatic solid tumors with mismatch repair defects, high microsatellite instability, and applied nivolumab approval for the treatment of colorectal cancer with a mismatch repair defect and high microsatellite instability.

However, although this colorectal cancer type with very good response to ICIs treatment, there may still exist some resistance and patients still have poor response to ICIs treatment. Romain Cohen, et al. analyzed 38 colorectal cancer patients diagnosed with MSI or dMMR and treated with ICIs and found five had ICB resistance, immediately after they reassessed the status of MSI or dMMR and found that three mCRC patients are characterized with MSS or pMMR (78) showing that MSI-H or dMMR mCRC is misdiagnosed into MSS or pMMR as MSI-H or dMMR status. Therefore, it appears crucial to make a correct diagnosis and to determine MSS and MMR status by immunohistochemistry or PCR before ICIs treatment. Besides, Carino Gurjao, et al. reported that a patient with MSI-H and extremely high neoantigen load found continued disease progression despite with ICIs treatment. Then they tested the intrinsic resistance of CRC by testing the genomic, transcriptomes, immunohistochemistry of patient tumors and related immune microenvironment. They found that the possible reasons for intrinsic resistance to MSI-CRC were biallelic deletion of B2M (associated with antigen presentation) and increasing infiltration of NK cells and M2 macrophage (79). Therefore, attempting to improve B2M biallelic deletion status and reducing NK cell and M2 macrophage infiltration are possible effective pathways to improve intrinsic resistance to MSI-CRC. Next, CRC patients with MSI-H or dMMR with BRAF mutations also have relatively poor prognosis (80–82), therefore, such patients should be specific analysis, and utilize different therapeutic targets or combination therapy may be needed to improve efficacy.

Unfortunately, CRC patients with MSI-H or dMMR who respond well to ICIs efficacy account for only 10–15% of the entire CRC patient population, and approximately 85–90% of CRC patients belongs to the MSS or pMMR type. The current general view is that mCRC tumor cells accompanied by pMMR or MSS features have reduced immunogenicity to CD8 + T cells, reduced tumor cell mutation load, reduced HLA class molecule expression, and missing B2M protein are the possible reasons for their poor or even no response to ICIs treatment. There is no doubt that it’s urgent to improve the efficacy of CRC patients with MSS or pMMR to significantly improve the quality of life of CRC patients.

It is widely accepted that pMMR or MSS mCRC tumor cells have reduced immunogenicity, lower mutational load, low expression of HLA class molecules, and loss of 2-heterococytoprotein may be the main reason of (83) for its low or non-response to ICIs treatment. An increasing quantity of researchers have invested basic and clinical research to improve ICIs treatment response in pMMR or MSS mCRC patients, in order to improve progression-free survival and improve their quality of life in CRC patients. They found that ICIs combined with Fruquitinib or Regorafenib (an antiangiogenic drug, the former combination works better than the latter and could significantly improve the progression-free survival of mCRC patients) (84), LDH-A inhibitors (85), MEK inhibitors (86, 87), celecoxib (an inhibitor of the cyclooxidase COX2) (88), TGF-β inhibitors (89), CXCR4 inhibitors (90) combined treatment of pMMR or MSS mCRC, could significantly improve the treatment response of such patients to ICB in response, which provides a vision of a promising cure for CRC, however, drugs partly produce some toxic side effects such as colitis and duodenal ulcer, one of the pathogenic factors in colorectal cancer. Therefore, there is an urgent need to explore an effective treatment of CRC patients, including MSI-H or dMMR characteristics and pMMR or MSS characteristics, to improve progression-free survival and improve their quality of life in CRC patients.

## m6A Epigenetic Modification

Current studies have shown that epigenetic defects played a crucial role in all malignant tumors with genetic defects (18, 19, 91, 92). Cancer is a disease caused by the continuous accumulation of genetic and epigenetic alteration. Generally, epigenetic alteration often precedes cancer and then induce gene mutations which lead to cancer (93). Almost all human cancer types contain epigenetic alterations. Because epigenetic modifications are reversible in nature (18–20), epigenetics has become an attractive target for cancer therapy. Recent studies have found that, in addition to being related to cancer progression, epigenetic modification is also a potential driver of drug resistance (17).

N6-methyladenosine (m6A) modification is the most common epigenetic methylation modification in RNA, such as mRNAs, ncRNAs, approximately 80% of all RNA methylation modification *in vivo* (94), which influences RNA function, for example, it has an great effect on RNA splicing, export, stability and translation. m6A modification is a reversible and dynamic in RNA epigenetic process. It is adjusted by m6A regulators, the main regulators containing “writer” (methyltransferase), “reader” (signal converter), combined with “eraser” (demethylation basease) (95).

**Figure 3:**
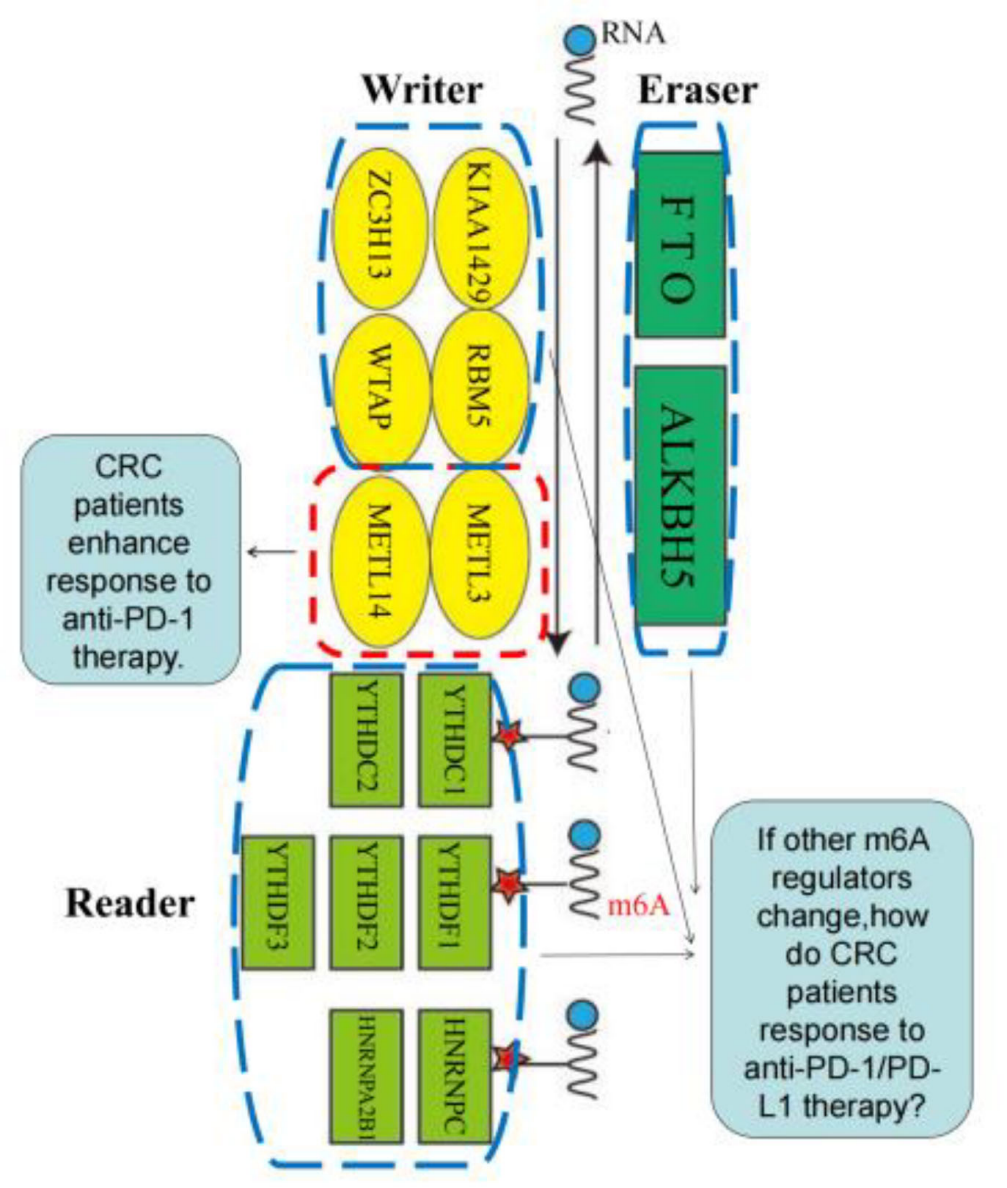
m6A regulators, including “writer” (methyltransferase), “reader” (signal converter) and “eraser” (demethylation basease).

Specifically, the writers, whose function is adding methylation into RNA to make the RNA methylated, up to date, are composed of METTL3, METTL14, WTAP, RBM15, RBM15B, ZC3H13 and KIAA1429 (96, 97). The readers, identifying methylation sites, are contained with YTHDC1, YTHDC2, YTHDF1, YTHDF2, YTHDF3, HNRNPC,IMP2 and HNRNPA2B1 (98–101). The erasers, whose role is deleting the methylation of RNA, mainly consist of FTO and ALKBH5 (102, 103) as revealed in **Figure 3**.

## m6A modification, ICIs IN CRC

At present, anti-immune checkpoint treatment of CRC is the thoroughly studied type of immunotherapy and has left some treating troubles so far (17). Researches lately have displayed that, in addition to being related to cancer progression, m6A epigenetic modification is also a potential driver of drug resistance (104). There are several main mechanisms that lead to anti-cancer drug resistance: changes in drug metabolism, deregulation of drug transport, mutations or changes in target protein expression, the above-mentioned mechanisms are the main reasons for the decrease in the efficacy of anti-cancer drugs (105–109).

Among several new methods to achieve the goal of drug sensitive, the regulation of m6A RNA modification has been demonstrated to be an overwhelming strategy for the drug resistance of various types of cancer cells. M6A regulates RNA’s different-stage function, such as RNA splicing, degradation and translation to take part in a variety of biological processes including cell proliferation, metabolism and metastasis, and the caused anti-tumor resistance (109).

Immune checkpoint inhibitors have been utilized to colorectal cancer therapy and provides a promising clinical advantage; however, it usually shows that CRC patients make a low response or resistance (110). To improve the efficacy of ICB, it is urgently needed to identify the underlying mechanism for the low-response or resistance of ICIs in CRC. Recent studies have shown that m6A modifications are highly related with the low-response or resistance of ICIs in CRC, one possible reason that m6A tightly connected with microenvironment of tumor and immune cells in CRC.

The m6A modification pathway is often misregulated in cancer (111). Bo Zhang, et al. revealed that m6A modification is necessary in TME (112–115). The high m6A score, characterized by decreased mutation burden and immune activation, is related to reduced neoantigen load and poor response to ICIs (115). Patients with higher m6A-related risks have lower expression of immune checkpoint molecules, indicating that ICIs treatment may be less efficient for this subgroup. Therefore, m6A risk characteristics could be used as immunotherapy predictors for STAD prognosis in a clinical setting, and the underlying mechanism of the relationship between immune checkpoint molecules and m6A methylation needs to be further studied (115). In the same way as gastric cancer, does this apply to the colorectal tumors belonging to the digestive tract tumor? This will provide new ideas for clinically predicting the ICIs response of patients with colorectal cancer and further strengthening ICIs therapeutic response in colorectal cancer.

### m6A Writers, ICIs in CRC

M6A modification regulated by METTL3 may promotes D.C. activation and maturation, Specific knockout Mettl3 in D.C. causes impaired phenotypic and functional maturation of D.C. and decreases their capacities of stimulating T cell responses (112). Specific depletion of Mettl3 in tumor-associated macrophages(TAM) resulted in CD8+ T cells dysfunction and tumor growth (113). With the reducing of m6A regulated by Mettl14, expression of the tumor suppressor KLF4 will be substantially increased and will further promote the migration of CRC cells and invasion (114). Interestingly some reseachers believed that m6A writers, such as Mettl3, WTAP, is positively associated with CRC cells invasion, migration, progression, and tumor stem cells with stemness and drug resistance (116–119).

Chen Xiaoxiang, et al. explored the function of m6A modification in CRC, illustrated the mechanism of m6A modification involved in the biological process of CRC, and confirmed that METTL14 is related to the progression of CRC *in vivo* and *in vitro* (112–114, 116–120). Lingling Wang et al. found that the deletion of methyltransferases Mettl3 and Mettl14 inhibited N6 methyladenosine (m6A) mRNA modification, enhanced pMMR-MSI-L colorectal cancer and melanoma patients’ response to anti-PD-1 therapy, and significantly slowed down tumor grows and prolongs the patient’s survival. In addition, the deficiency of MettL3 or Mettl14 in tumors results in enhancing cytotoxic tumor-infiltrating CD8+ T cells, and increasing the secretion of IFN-c, Cxcl9 and Cxcl10 in TME *in vivo*, which proves that the immune system and tumor microenvironment have metastasized in tumor m6A mRNA. Changes will occur after the enzyme is removed. In patients with colorectal cancer and melanoma undergoing immunotherapy, changes in the transcriptome profile of methyltransferase-deficient tumors in tumor cells indicate that the activation of IFN-c signaling is the key to resensitization, while external transcriptome analysis shows that IFN -c-Stat1-Irf1 axis transcript lacking m6A modification contributes to the stabilization of m6A reader Ythdf2-mediated transcripts, thereby explaining the up-regulation of IFN-c signaling and changes in the tumor microenvironment (121), this discovery will further promote the understanding and in-depth study of m6A methyltransferase in the anti-PD-1/PD-L1 treatment of colorectal cancer.

However, whether and how m6A modifications writers alteration influence the efficiency of ICIs in colorectal cancer, still remains further research.

### m6A Erasers, ICIs in CRC 

Zeyan Zhang, et al. studied m6A erasers and found that reducing FTO expression, inhibiting MZF1 expression, and thus c-Myc expression and hindered CRC cell proliferation and progression (122). Contrary to the conclusion, Danyun Ruan, et al. and Sebastien Relier, et al. suggested that inhibiting FTO expression would promote CRC metastasis, poor prognosis and high recurrence in CRC patients (101, 123).

The study by Seungwon Yang, et al. found that under the action of the demethylase FTO, m6A will promote the growth of melanoma and reduce its response to anti-PD-1 blocking immunotherapy. When FTO is knocked out, it can increase m6A methylation in key tumorigenic melanoma cells (including PD-1, CXCR4 and SOX10), resulting in increased RNA attenuation through m6A reader YTHDF2, and then cause that melanoma cells are sensitive to interferon-γ (IFN-γ) and make melanoma sensitive to anti-PD-1 treatment. It is generally believed that inhibiting the combination of FTO and anti-PD-1 blockade may attenuate the resistance of melanoma to immunotherapy and improve the treatment response (124). However, they did not conduct research on colorectal cancer like Lingling Wang et al., but this will open a new way of thinking about ICIs resistance in colorectal cancer patients.

Then the study by Na Lia, et al. found that the knockout of the m6A demethylase ALKBH5 would make tumors susceptible to cancer immunotherapy. Specifically, ALKBH5 regulates the expression level and lactic acid content of Mct4/Slc16a3 in the TME and the constitution of tumor infiltrating Tregs and myeloid-derived suppressor cells. The research results show that m6A demethylase in tumor cells contributes to the effect of immunotherapy, and ALKBH5 is identified as a potential therapeutic target to improve the efficacy of immunotherapy for melanoma, colorectal cancer and other potential cancers (125).

However, whether and how m6A modifications erasers variations influence the efficiency of ICIs in colorectal cancer, still remains further research.

### m6A Readers, ICIs in CRC

Numerous studies on m6A Reader have confirmed four genes (YTHDF1, IGF2BP1, IGF2BP1, IGFBP3, EIF3B) is a potential biomarker of CRC. It was found to downregulate YTHDF1 or IMP2, can further regulate the GLS1-glutamine metabolic axis, improve the stability of the m6A-modified GSK3β mRNA, inhibition of Wnt/-catenin/cyclin D1 expression, inhibition of CRC cell proliferation, colony formation, and increase the apoptosis levels in CRC cells, To sensitized cisplatin-resistant CRC cells (126–128).

Kazuo Tsuchiya, et al. used immunohistochemistry to detect the protein expression levels of m6A readers, such as YTHDF1 and YTHDF2 in 603 cases of non-small cell lung cancer tissues and evaluated four subgroups as TILs in tumor nests and surrounding stroma (PD-1, CD8, Foxp3 and CD45RO), and to study the differential expression of PD-L1 in lung cancer cells lacking YTHDF1 and YTHDF2. They found that YTHDF1 and YTHDF2 lowerly expressed in advanced tumors than that of early tumors, and the expression of YTHDF1 and YTHDF2 were also advised to be independent favorable prognostic factors for recurrence-free survival. In tumors with high expression of YTHDF1 and YTHDF2, the TIL density of almost all four lymphocyte subgroups in the stroma was significantly increased. *In vitro*, YTHDF1 and YTHDF2 knockout in cells up-regulated the expression of tumor PD-L1 and changed a variety of immune-related genes. It shows that the high expression of YTHDF1 and YTHDF2 is related to the good prognosis of NSCLC patients, the increase in the number of TIL and the down-regulation of PD-L1. YTHDF1 and YTHDF2 may be new prognostic and drug targets related to lung cancer tumor immune microenvironment (129). However, whether the up-regulation of YTHDF1 and YTHDF2 is effective for patients with colorectal cancer needs further research.

However, whether and how m6A modifications readers changes influence the efficiency of ICIs in colorectal cancer, still remains further research.

The possible reasons for such interestingly different and even opposite conclusions for different researchers in the same study subject maybe depended on m6A modified different RNA sites, different patterns in different immune cells, or different types of CRC, for example, MSS/pMMR or MSI-H/dMMR and whether accompanied by BRAF mutations.

However, due to methodological limitations, these studies are limited to one or two RNA modification “writers”, “erasers” or “readers”, but the antitumor effects of RNA modifications are characterized by highly complicated interactions of many regulators. Thus, a comprehensive and full understanding of how the regulatory networks of multiple RNA modification, there is no doubt that, will absolutely help us to have a better understanding of immunomodulatory and development of immunotherapy strategies in low-response or resistance of ICIs in CRC patients.

## Conclusions and Prospects

The success of tumor immunotherapy, especially the success of ICIs treatment, has brought encouraging hope and confidence for better treatment and possible cure of colorectal cancer patients. However, because about 85-90% of colorectal cancer patients have the characteristics of pMMR or MSI-L in clinical practice, whose typical feature is that the tumor with a lower mutation burden has a poor curative effect, usually showing resistance to ICIs treatment. So this will force us to further explore the cure of colorectal cancer and the mechanism of PD-1/PD-L1 resistance.

In this review, we summarized the mechanism of immune escape in the process of general cancer and the current status of immune checkpoint blockade treatment of colorectal cancer. At the same time, we also deeply explored and guess the role of m6A in mechanism of resistance in colorectal cancer anti-PD-1/PD-L1 therapy.

For the brief mechanism of m6A to relieve the resistance of colorectal cancer patients to anti-PD-1/PD-L1 therapy, we summarize the existing research and speculate the following findings. The m6A modification regulated by the m6A regulator includes the “writer” (methyltransferase_METTL3, METL14), “reader” (signal converter_YTHDC1, YTHDC2) and “eraser” (demethylase_FTO, ALKBH5) (67). Inhibition of “writers” (methyltransferase_Mettl3, Mettl14) can inhibit m6A modification, enhance the response of pMMR-MSI-L colorectal cancer patients to anti-PD-1 treatment, and significantly slow down tumor growth and prolong patient survival; activation the high expression of “readers” (signal converters_YTHDF1 and YTHDF2) may be related to the good prognosis of patients with colorectal cancer, the increase in the number of TIL, and the down-regulation of PD-L1, and may become a novel related to the immune microenvironment of colorectal cancer. The prognosis and drug targets of the drug; inhibiting the “eraser” (demethylase_FTO, ALKBH5) may increase the sensitivity of colorectal cancer patients to immune checkpoint therapy and reduce resistance after the anti-PD-1/PD-L1 therapy.

At present, in colorectal cancer ICIs treatment resistance research, only Lingling Wang et al. from the m6A “author” (methyltransferase_Mettl3, Mettl14) point of view, found that the deletion of methyltransferase Mettl3 and Mettl14 inhibits N6 methyladenosine (m6A) mRNA modification, enhances the response of pMMR-MSI-L colorectal cancer and melanoma patients to anti-PD-1 treatment, and significantly slows tumor growth and prolongs patient survival. And whether m6A “reader” (signal converter_YTHDF1, YTHDF2), “eraser” (demethylase_FTO, ALKBH5), etc. or how to convert can increase the sensitivity of colorectal cancer patients to immune checkpoint therapy and reduce the resistance of colorectal cancer patients to anti-PD-1/PD-L1 therapy still needs further research.

## Author Contributions

HT, HW, and AS made significant contribution on writing this review. JH designed the topic and checked the manuscript. All authors contributed to the article and approved the submitted version.

## Conflict of Interest

The authors declare that the research was conducted in the absence of any commercial or financial relationships that could be construed as a potential conflict of interest.

## Publisher’s Note

All claims expressed in this article are solely those of the authors and do not necessarily represent those of their affiliated organizations, or those of the publisher, the editors and the reviewers. Any product that may be evaluated in this article, or claim that may be made by its manufacturer, is not guaranteed or endorsed by the publisher.
